# General population’s perceptions regarding marriage in patients with Bipolar disorder

**DOI:** 10.1192/j.eurpsy.2025.1091

**Published:** 2025-08-26

**Authors:** Y. Trabelsi, B. N. Saguem, N. Jaafar

**Affiliations:** 1 Psychiatry Department, Farhat Hached Hospital, sousse, Tunisia

## Abstract

**Introduction:**

The general population’s views on marrying individuals with bipolar disorder are shaped by a combination of personal experiences, cultural beliefs and media portrayals, and this emphasize the challenges for successful, supportive relationships among them.

**Objectives:**

To explore these perceptions we performed a qualitative analysis of public attitudes toward marriage in individuals with bipolar disorder.

**Methods:**

A cross-sectional study was carried out using a convenience sampling approach among the general population. The survey was distributed through social media platforms and, in addition to collecting socio-demographic and clinical data, it provided a detailed account of the symptoms and outcomes associated with bipolar disorder. It also included open-ended questions aimed to evaluate perceptions of the potential advantages and disadvantages of being in a relationship with someone who has bipolar disorder. The responses were analyzed, and common themes reflecting public opinion were identified.

**Results:**

A total of 304 participants, mostly aged between 20 and 30, were included, with women making up 80.9% of the group. The majority held a university degree. A family history of psychiatric conditions was reported by 35.6% of the participants, and 23.35% mentioned living with someone who has a psychiatric disorder. Additionally, around 87% of the participants acknowledged having consulted a psychiatrist at least once in their lifetime.

Responses regarding potential disbenefits of being married to a patient with bipolar disorder included 9 themes. The most representative ones were 1) the fear of dealing with mood swings and recurrent mood episodes (10.2%); 2) the impossibility of having a stable relationship (7.2%), 3) the risk of physical and/or verbal violence (6.4%); 4) the elevated risk of separation (4.6%); and 5) the risk of suicide (3.9%). Other themes included concerns about 6) transmitting the pathology to their descendants; 7) dealing with a partner who refuses treatments; 8) having an irresponsible companion; and 9) facing financial problems.

About 8.9% of the participants stated that they were willing to get married with a patient with bipolar disorder in order to help him survive his illness; 6.2% of them qualified him as intelligent and affectionate; and 5.2% considered manic episodes as an opportunity to alter routine.

**Conclusions:**

Public perceptions of marriage with individuals with bipolar disorder are shaped by a complex interplay of stigma, fear, and misinformation. While negative stereotypes are prevalent, there is also a recognition that with the right support, successful marriages are possible. Addressing the knowledge gaps and promoting empathy are crucial steps in reducing stigma and supporting individuals with bipolar disorder in their personal and marital lives.

**Disclosure of Interest:**

None Declared

